# Importance of the latex-clearing protein (Lcp) for poly(*cis*-1,4-isoprene) rubber cleavage in *Streptomyces* sp. K30

**DOI:** 10.1002/mbo3.3

**Published:** 2012-03

**Authors:** Meral Yikmis, Alexander Steinbüchel

**Affiliations:** 1 Institut für Molekulare Mikrobiologie und BiotechnologieWestfä lische Wilhelms-Universitä t Münster D-48149, Münster Germany; 2 King Abdulaziz UniversityJeddah, Saudi Arabia

**Keywords:** Biopolymer, knock out *lcp* mutant, *lcp* (latex-clearing protein), natural rubber latex, poly(*cis*-14-isoprene) rubber degradation, secretion, *Streptomyces*

## Abstract

*Streptomyces* sp. strain K30 induces the formation of an extracellular Lcp (latex-clearing protein) during poly(*cis*-1,4-isoprene) degradation. To investigate the function of this enzyme in *Streptomyces* sp. strain K30, the *lcp* gene was disrupted. This was the first time that the screening for a knock out *lcp* mutant of *Streptomyces* sp. strain K30 was successful. The resulting mutant *Streptomyces* sp. K30_*lcp*ΩKm exhibited reduced growth in liquid mineral salts media containing poly(*cis*-1,4-isoprene) as the sole carbon and energy source. Additionally, there was no detectable Lcp activity on latex overlay agar plates. When Lcp from *Streptomyces* sp. strain K30 was heterologously expressed in strains TK23 and TK24 of *Streptomyces lividans* and a strain of *S. erythraea* with plasmid pIJ6021::*lcp*, the recombinant strains acquired the ability to cleave synthetic poly(*cis*-1,4-isoprene), confirming the involvement of Lcp in initial polymer cleavage. Specific anti-LcpK30 IgGs were employed in Western blot analysis to detect the secretion of Lcp in the supernatant. We have conducted an important experiment to demonstrate Lcp activity using the supernatant of these Lcp-expressing strains in vitro. All three strains obviously secreted a functional Lcp, as indicated by the formation of halo. Functional testing of Lcp with different plasmids in *Escherichia coli* strains and *Pseudomonas* strains was, however, not successful.

## Introduction

Actinomycetes play a major role in the degradation of natural rubber (NR), while some other bacteria and fungi are also known to attack rubber (Kumar et al. 1983). Microorganisms capable of degrading NR cannot degrade synthetic rubbers other than synthetic isoprene rubber (Linos and Steinbüchel 1998). The latex-clearing protein (Lcp) from the rubber-degrading bacterium *Streptomyces* sp. strain K30 is involved in the initial cleavage of poly(*cis*-1,4-isoprene), yielding isoprenoid aldehydes and ketones (Rose et al. 2005). Lcp homologues have so far been detected in all investigated clear zone forming rubber-degrading bacteria.

The microbial degradation of natural and synthetic poly(*cis*-1,4-isoprene) rubber is currently being intensively investigated (Rose et al. 2005; Rose and Steinbüchel 2005), and two different strategies for the degradation of isoprene rubber have been unraveled thereby distinguishing two different groups of rubber-degrading bacteria (Peczynska-Czoch and Mordarski 1988).

Members of the first group form translucent halos when cultivated on solid media containing dispersed latex particles, indicating the excretion of rubber-cleaving enzymes. Mycelium-forming actinomycetes such as *Actinoplanes*, *Micromonospora*, and *Streptomyces* species belong to this group. The second group comprises mycolic acid containing *Actinobacteria* belonging to the genera *Gordonia*, *Mycobacterium*, and *Nocardia*. These bacteria do not form translucent halos, but they grow adhesively on the surface of rubber particles in liquid culture, and they represent the most potent rubber-degrading bacterial strains (Arenskötter et al. 2004). *Xanthomonas* sp. strain 35Y is the only known rubber-degrading bacterium that does not belong to the actinomycetes but is a Gram-negative bacterium (Jendrossek and Reinhardt 2003).

A rubber oxygenase RoxA, which is synthesized during growth on NR latex by *Xanthomonas* sp. 35Y, was identified (Jendrossek and Reinhardt 2003; Braaz et al. 2005). This bacterium is strictly aerobic and produces insoluble yellow pigments in the cell. *Xanthomonas* species belong to the phylum Proteobacteria and stain Gram-negative. However, regarding the strategy of rubber degradation, it belongs to the first group and forms halos on rubber-containing agar plates.

**Figure 1:**
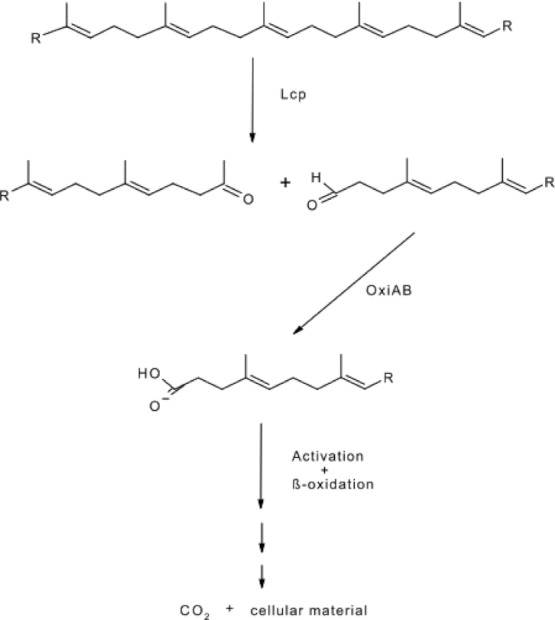
Hypothetical pathway of poly(*cis* -1,4-isoprene) degradation by *Streptomyces* sp. strain K30.

In a hypothetical pathway supposed for rubber degradation, Bode et al. (2000) postulated a not further characterized oxidation of the degradation product acetonyldiprenylacetoaldehyde to the corresponding acid. This aldehyde compound was previously also identified by Tsuchii and Takeda (1990) after incubation of NR with *Xanthomonas* sp. 35Y and subsequent ether extraction. This oxidation step converting the aldehyde to the corresponding acid could possibly be performed by an enzyme similar to OxiAB whereas Lcp is responsible for the first step in this pathway, the oxidative cleavage of the polyisoprene backbone. These aldehyde and ketones with low molecular weights, which are then possibly further oxidized by OxiAB to the corresponding acids, are activated and metabolized via the β-oxidation pathway in *Streptomyces* sp. K30 (Fig. 1).

Rose et al. (2005) identified the *lcp* gene encoding a latex clearing protein from *Streptomyces* sp. strain K30. The clear zone forming phenotype was used to identify clones harboring the *lcp* gene from *Streptomyces* sp. strain K30 by phenotypic complementation of a clear zone negative mutant. The 1191-bp structural gene was preceded by a putative signal sequence and restored the capability of forming clear zones on NR latex agar plates in the mutant. Like RoxA, also Lcp is secreted into the extracellular medium leading to the formation of translucent halos on NR latex. However, both proteins share no sequence homologies. The putative translation product of *lcp* exhibited strong homologies (50% aa identity) to a putative secreted protein from *S. coelicolor* strain A3 (Bagdasarian and Timmis 1982), which is another clear zone forming strain (Rose et al. 2005). Sequence analysis of Lcp and characterization of mutants of *Streptomyces* sp. strain K30 showed secretion of Lcp via the twin-arginine translocation (Tat) pathway (Yikmis et al. 2008; Thomas et al. 2001).

Because expression of functional Lcp in recombinant *Escherichia coli* strains or in recombinant γ-Proteobacteria such as *Pseudomonas putida* was not successful, expression of recombinant Lcp in other bacteria belonging to the genus *Streptomyces* sp., was performed. In this study, we show a system optimized for the expression of recombinant Lcp and the microbial degradation of rubber by these strains. Three actinomycetes strains, *S*. *lividans* TK23, TK24, and *Saccharopolyspora erythraea*, were able to produce clear zones on rubber overlay agar plates upon transfer of the wild-type *lcp* gene to these strains. Furthermore, we have conducted an important experiment to demonstrate Lcp activity using the supernatant of these Lcp-expressing strains in vitro. All three strains obviously secreted a functional Lcp, as indicated by the formation of a halo. We also generated a knock out *lcp* mutant from *Streptomyces* sp. strain K30 to characterize the role of Lcp with regard to poly(*cis*-1,4-isoprene) rubber degradation. By isolating and investigating the knock out *lcp* mutant, we have now confirmed evidence that Lcp is responsible for the initial rubber degradation.

## Materials and Methods

### Bacterial strains and culture conditions

Bacteria and plasmids used in this study are listed in Table 1. If not otherwise mentioned, cells of *Streptomyces* sp. were grown in tryptic soy broth (TSB) medium at 30°C (Merck, Darmstadt, Germany), whereas cells of *E. coli* were cultivated at 37°C in Luria Bertani broth (LB) (Sambrook et al. 1989), mineral salts medium (MSM) (Schlegel et al. 1961), or in standard I (St-I) medium (Merck). Antibiotics were applied according to Sambrook et al. (1989) and as indicated in the text. For growth experiments with natural and synthetic polyisoprene, cells were cultivated in MSM (Schlegel et al. 1961). The following carbon sources were added to liquid MSM: 0.5% (v/v) natural latex concentrate (Neotex Latz; Weber & Schaer, Hamburg, Germany) or 0.3% (w/v) synthetic poly(*cis*-1,4-isoprene) with an average molecular mass of 800 kDa. Liquid cultures were grown in Erlenmeyer flasks, which were incubated on a horizontal rotary shaker. Solid media were prepared by addition of agar–agar (18 g/L). Purified NR latex from *Hevea brasiliensis* was a gift from Weber & Schaer and was used for the preparation of overlay plates as described previously (Jendrossek et al. 1997). Latex overlay agar plates were used for growth of clear zone forming strains. For this, MSM agar plates were covered with an overlay of MSM agar containing 0.2% (v/v) disperged latex concentrate.

**Table 1:** Bacterial strains, plasmids, and oligonucleotides used in this study

Strains and plasmids	Relevant characteristics	Reference
**Strains**		
*Streptomyces* sp. K30	Wild type producing clear zones on NR latex overlay agar plates	Rose et al. 2005
*Streptomyces* sp. K30_ *lcp* ΩKm	*lcp* knock out mutant, clear zone negative	This study
*Streptomyces lividans* TK23	Clear zone negative; host strain for heterologous expression	Hopwood 1983
*Streptomyces lividans* TK23 pIJ6021:: *lcp*	Producing clear zones on natural latex overlay agar plates; host strain for heterologous expression harboring wild-type *lcp* from *Streptomyces* sp. strain K30	This study
*Streptomyces lividans* TK24	Clear zone negative; host strain for heterologous expression	Hopwood 1986
*Streptomyces lividans* TK24 pIJ6021:: *lcp*	Producing clear zones on natural latex overlay agar plates; host strain for heterologous expression harboring wild-type *lcp* from *Streptomyces* sp. strain K30	This study
*Saccharopolyspora erythraea*	Wild type, clear zone negative; host strain for heterologous expression	DSMZ 40517
*Saccharopolyspora erythraea* pIJ6021:: *lcp*	Producing clear zones on natural latex overlay agar plates; host strain for heterologous expression harboring wild-type *lcp* from *Streptomyces* sp. strain K30	This study
*Pseudomonas putida*	Wild type, clear zone negative; host strain for heterologous expression	DSMZ 291
*Pseudomonas putida* KT2440	Clear zone negative; host strain for heterologous expression; pWW0-, r-, m+; spontaneous mutant from *P. putida* mt-2	Bagdasarian et al. 1982
*Pseudomonas putida* KT2440 pBBR1MCS2::Lcp_His6	Producing clear zones on natural latex overlay agar plates; host strain for heterologous expression harboring wild-type *His* - *tagged lcp* protein from *Streptomyces* sp. strain K30	This study
*Pseudomonas putida* KT2440 pJB653::Lcp_His6	Producing clear zones on natural latex overlay agar plates; host strain for heterologous expression harboring wild type *His* - *tagged lcp* protein from *Streptomyces* sp. strain K30	This study
*Pseudomonas putida* KT2440^StrR^	Clear zone negative; host strain for heterologous expression; pWW0-, r-, m+; spontaneous streptomycin resistant mutant from *P. putida* mt-2	Bagdasarian et al. 1981
*Pseudomonas putida* KT2440^StrR^ pBBR1MCS2::Lcp_His6	Producing clear zones on natural latex overlay agar plates; host strain for heterologous expression harboring wild-type *His* - *tagged lcp* protein from *Streptomyces* sp. strain K30	This study
*Pseudomonas putida* KT2440^StrR^ pJB653::Lcp_His6	Producing clear zones on natural latex overlay agar plates; host strain for heterologous expression harboring wild-type *His* - *tagged lcp* protein from *Streptomyces* sp. strain K30	This study
*Escherichia coli* Top10	Donor strain	Stratagene
*Escherichia coli* ET12567	Nonmethylating plasmid donor strain	Flett and MacNeil 1992
**Plasmids**		
pET23a:: *lcp* _1	pET23a harboring the wild-type *lcp* from *Streptomyces* sp. strain K30	This study
pBBR1MCS2	Broad host-range promoter-probe vector, pBBR1MCS2	Kovach et al. 1995
pBBR1MCS2::Lcp_His6	Shuttle vector harboring the wild-type *His-tagged lcp* protein from *Streptomyces* sp. strain K30	This study
pJB653	Broad host-range promoter-probe vector, pJB653	Blatny et al. 1997
pJB653::Lcp_His6	Shuttle vector harboring the wild-type *His-tagged lcp* protein from *Streptomyces* sp. strain K30	This study
pGEM-T Easy	*E. coli* TA cloning vector; Ap^r^	Promega
pIJ6021	High-copy-number plasmid expression vector; contains a thiostrepton-inducible promoter, *P_tipA_*, from *Streptomyces lividans* 66	Takano et al. 1995
pIJ6021:: *lcp*	pIJ6021 harboring wild-type *lcp* from *Streptomyces* sp. strain K30	This study
pIJ702	Plasmid contains the tyrosinase gene and thiostrepton resistance (*tsr*) gene	Rose et al. 2005
pIJ702:: *lcp* _1	pIJ702 harboring wild-type gene and the native promoter region of *lcp* isolated from *Streptomyces* sp. strain K30	This study
pIJ702:: *lcp*	pIJ702 harboring the wild-type *lcp* from *Streptomyces* sp. strain K30	Rose et al. 2005
**Oligonucleotides**		
PSPNter	CCGAGATCTCGGCAGGACGAACTCCCCG	Rose et al. 2005
PSPCter	CCGAGATCTGGTGCGTCGAGG	Rose et al. 2005
Hya_FW_XbaI	AATCTAGAAATAATTTTGTTTAACTTTAAGAAGGAGATATACATATGAACAACGAAGAAACCTTTTATCAGGCC	This study
Hya_RW_NcoI	AACCATGGGCGCCCACGCAATTTTCGGC	This study
pqspBBR-for:_Sal	ATATGTCGACCTAAAATGGAGTCATGAACAACGAAGAAAACCTTTTATCAGGCCATG	This study
pqspBBR-rev:_Sac	ATATGAGCTCCACCACCATCACCACCATGCTCGGACGGTTCACATCCGGAATATCAATCG	This study
pqspJB-for:_Sbf	ATATCCTGCAGGTAAGGAGTCATGAACAACGAAGAAACCTTTTATCAGGCCATG	This study
pqspJB-rev:_Sac	TATAGAGCTCCACCACCATCACCACCATGCTCGGACGGTTCACATCCGGAATATCAATCG	This study
Lcp_EcoRI_6021	AAAGAATTCTCAGGACGGGCGGTTGACGTCCGGGGATG	This study
Lcp_NdeI_6021	AAAAAAACATATGGCGATCCGCCTTCCGCCCGGCGCCCCGCG	This study
N_Lcp	GGATCCTTACGTCAGTAGGCGTGGTCCAGGCCGTCGGTCGG	This study
C_Lcp	GGATCCCGACCGGGATGACGTGCGGCAGTGGGCCC	This study

### Protoplast formation and regeneration

Protoplasts of *Streptomyces* sp. were prepared from cells grown in modified YEME (3%, w/v, yeast extract; 5%, w/v, Bacto peptone; 3%, w/v, malt extract; 34%, w/v, sucrose) medium (Kieser et al. 2000). R5 agar plates were used for protoplast regeneration (Kieser et al. 2000).

### Isolation, analysis, and manipulation of DNA

Plasmid DNA was prepared from crude cell lysates by the alkaline extraction method (Kieser et al. 2000). Cells of *Streptomyces* were incubated at 37°C for lysis in presence of lysozyme (2 mg/mL) for at least 2 h. Recombinant DNA techniques in *Streptomyces* were performed as described by Kieser et al. (2000). Total DNA from *Streptomyces* was isolated by the versatile quick-prep method for Gram-positive bacteria according to Pospiech and Neumann (1995). DNA was restricted with endonucleases (Gibco/BRL, Gaithersburg, MD) as mentioned in the text under the conditions recommended by the manufacturer. All other genetic procedures and manipulations were conducted as described by Sambrook et al. (1989).

### Aldehyde staining of poly(*cis*-1,4-isoprene) and degradation products

Aldehyde groups resulting from poly(*cis*-1,4-isoprene) cleavage during clear zone formation on NR latex overlay agar plates were stained for 20 min with Schiff's reagent. Afterwards, the staining reagent was removed, and the slides were washed with sulfite solution. The composition of the staining solution was as follows: 2 g of fuchsin dissolved in 50 mL of glacial acetic acid, 10 g Na_2_S_2_O_5_, 100 mL of 0.1 N HCl, and 50 mL H_2_O. The composition of the sulfite solution was 5 g of Na_2_S_2_O_5_ plus 5 mL of concentrated HCl (37–38%, v/v) in a 100-mL aqueous solution.

### DNA sequencing and sequence analysis

DNA sequencing was carried out at the Institut für Klinische Chemie und Laboratoriumsmedizin (Münster, Germany). Obtained sequences were analyzed using Genamics Expression software (version 1.100 [http://genamics.com/expression/index.htm]). Sequence comparisons and alignments were performed using the BLAST online service available on NCBI (National Center for Biotechnology Information [http://blast.ncbi.nlm.nih.gov/Blast.cgi]), BioEdit (Hall 1998), and ClustalW (Towbin et al. 1979). Postgenome analyses were made using the KEGG (Kyoto encyclopedia of genes and genomes) database at GenomeNet (Kanehisa 1997; Kanehisa and Goto 2000; Kanehisa 2002; Kieser et al. 2000, [http://www.genome.jp]).

### Cloning and expression of Lcp

The coding region of *lcp* from *Streptomyces* sp. K30 was amplified by PCR by applying primers Lcp_EcoRI_6021 and Lcp_NdeI_6021. The amplified PCR product was then cloned into the pGEM-T Easy vector, excised by restriction with *Eco*RI and *Nde*I, and ligated to *Eco*RI-*Nde*I-linearized plasmid pIJ6021 DNA. For expression analyses, the resulting plasmid, pIJ6021::*lcp*, was transferred to *Streptomyces* strains via protoplast transformation (Hidalgo et al. 2004). These strains were cultivated in LB medium containing antibiotics, which were applied according to Sambrook et al. (1989), at 30°C on a rotary shaker at 180 rpm. After 48 h of incubation, the cells were harvested by centrifugation (20 min, 4°C, 4000 rpm; Megafuge 1.0R, HERAEUS SEPATECH GMBH, Osterode, Germany). The resulting supernatant was used for further characterization by SDS-polyacrylamide gel electrophoresis (PAGE).

### Preparation of cell-free extracts

Supernatants from 250-mL cell suspensions were concentrated by ultrafiltration (VIVASCIENCE, Satorius Group, Göttingen, Germany) to a volume of 1 mL. For further characterization, the samples were diluted in gel loading buffer (1%, w/v, SDS; 1.25%, w/v, β-mercaptoethanol; 0.25 mM EDTA; 10%, v/v, glycerol; 0.001%, w/v, bromophenol blue; 12.5 mM Tris-HCl, pH 6.8), denaturated for 10 min at 95°C, and separated in a preparative SDS-PAGE gel (12%, w/v, polyacrylamide) using a PrepCell 491 apparatus (BIO-RAD, Richmond, CA).

### SDS-PAGE and Western blot analysis

Samples were resuspended in gel loading buffer (0.6%, w/v, SDS; 1.25%, w/v, β-mercaptoethanol; 0.25 mM EDTA; 10%, v/v, glycerol; 0.001%, w/v, bromophenol blue; and 12.5 mM Tris-HCl, pH 6.8). Proteins were prepared as described by Laemmli (1970) and were stained with Coomassie brilliant blue R-250 (Weber and Osborn 1969). Proteins blotted from SDS-polyacrylamide gels onto nitrocellulose BA83 membranes (pore size, 0.2 mm; Schleicher & Schuell, Dassel, Germany) were analyzed immunologically as described by Hein et al. (1998). To determine the N-terminal amino acid sequence, the proteins were blotted from an SDS-polyacrylamide gel onto a polyvinylidene difluoride membrane (Millipore, Bedford, MA) according to the method of Towbin et al. (1979) by use of a Semidry Fast Blot B33 apparatus and were analyzed by automated Edman degradation.

### Expression of 6xHis-tagged Lcp in *E. coli* strain BL21(DE3), isolation of inclusion bodies, and generation of anti-LcpK30 antibodies

*Escherichia coli* strain BL21(DE3) harboring plasmid pET-23a::lcp His was cultivated in LB medium at 37°C to an OD600 of 0.5, and then expression was induced by addition of IPTG to a final concentration of 1 mM for 3 h yielding cells with inclusion bodies (IBs). For isolation of IBs, the cells of a 100-mL culture were harvested, resuspended in 4 mL 20 mM Tris-HCl (pH 8.0) buffer, and disrupted by a twofold French press passage at 1000 MPa. The disrupted cells were centrifuged at 25,000 *g* for 15 min at 4°C. The obtained pellet was resuspended in 3 mL cold IB wash buffer (2 M urea, 20 mM Tris-HCl, 0.5 M NaCl, 2% Triton X-100, pH 8.0) by sonication (1 min/mL with an amplitude of 40 μm) with a Bandelin Sonopuls GM200 ultrasonic disintegrator. After 15 min centrifugation at 4°C and 25,000 *g*, treatment with IB wash buffer, resuspension by sonication, and centrifugation were repeated for three times. The purified IBs were dissolved in SDS denaturation buffer (Laemmli 1970). A sample, consisting of the dissolved IBs containing the extracted Lcp protein, was separated by SDS-PAGE, excised from the gel, and its identity was confirmed by MALDI-TOF analysis (Bröker et al. 2008), before it was used for generation of polyclonal antibodies in rabbits in custom by “Eurogentec” (Seraing, Belgium). Purified polyclonal rabbit anti-LcpK30 IgGs were obtained from the serum by chromatography on Protein A-Sepharose (Hjelm et al. 1972).

### Immunoblotting

Protein detection was performed with anti-Lcp antibodies. PVDF (Polyvinylidene Difluoride) membranes with blotted proteins were placed for 1 h in skim milk (5%, w/v) to block nonspecifically binding domains. After a membrane was washed with Tris-buffered saline (TBS) Tween buffer (50 mM Tris-HCl [pH 7.5], 150 mM NaCl, 0.025% [v/v] Tween 20), it was incubated in an antibody solution (antibodies diluted 1:2000 in TBS-Tween buffer; 200 cm^−2^ membrane) and shaken overnight at room temperature. The membrane was washed three times for 10 min with TBS-Tween and was then incubated with secondary (alkaline phosphatase conjugated goat anti-rabbit immunoglobulin [IgG] [Sigma-Aldrich GmbH, Munich, Germany] diluted 1:2000 in TBS-Tween buffer; 200 μl cm^−2^ membrane) and shaken for 1 h at room temperature. The membrane was then washed three times for 10 min with TBS-Tween buffer and stained using 5-bromo-4-chloro-3-indolylphosphate (BCIP)/nitroblue tetrazolium tablets dissolved in 10-mL H_2_O (Sigma, Deisenhofen, Germany).

### Determination of mineralization

Evidence for biodegradation of the poly(*cis*-1,4-isoprene) hydrocarbon chain to CO_2_ was obtained by determination of CO_2_ evolution during aerobic cultivation of cells in presence of poly(*cis*-1,4-isoprene) as the sole carbon source. Determination was carried out in tightly closed Erlenmeyer flasks by using the property of Ba(OH)_2_ to precipitate CO_2_ as BaCO_3_. The flasks, containing 50-mL MSM, the rubber substrate [latex concentrate or poly(*cis*-1,4-isoprene)], and a test tube containing 15 mL of a 0.2 M Ba(OH)_2_ solution, were inoculated with 0.3% (v/v) of a well-grown culture. At each measurement point, the flasks were aerated, and the test tubes were replaced by new tubes containing fresh Ba(OH)_2_ solution. Consumption of carbonate by precipitation of CO_3_^2−^ as BaCO_3_ was determined for each period by titration with HCl and was compared to that of a noninoculated control. The mineralization rate was calculated as follows: mineralization (% CO_2_) = (required amount HCl [mL] × 0.252 M)/(C content of applied amount of *cis*-1,4-polyisoprene [mmol]) × 2.

## Results and Discussion

### Heterologous expression of *lcp* in *E. coli*

We previously identified Lcp as an important gene required for rubber degradation by *Streptomyces* sp. strain K30 (Rose et al. 2005) and aimed to characterize the heterologous expression of the gene product in the present study. For functionally and detailed characterization of the secretion–expression of *lcp* from *Streptomyces* sp. K30, the gene *lcp* was amplified employing the primers Hya_FW_XbaI and Hya_RW_NcoI (Table 1), and the PCR product was subsequently cloned into the *Xba*I and *Nco*I site of pET23a yielding pET23a::*lcp*. Additionally, *lcp* was subcloned into plasmids pUC19 and pET19b. However, the expression of all these different recombinant plasmids in several *E. coli* strains resulted in an overproduction of an inactive Lcp protein. However, despite of applying various experimental conditions such as cultivating the cells in LB medium or MSM, the protein was not active. In addition, different incubation temperatures (37°C, 28°C, or 20°C) with high or slow shaking rates of the culture vessels were tested, however, also here, no *E. coli* transformant showed an active Lcp protein, which allowed further analysis (data not shown). *Escherichia coli* was therefore not suitable to study the expression of Lcp.

### Heterologous expression of lcp in *Pseudomonas*

After due consideration, we constructed hybrid plasmids for gene cloning in the metabolically versatile bacterial genus *Pseudomonas* (Regenhardt et al. 2002). *Pseudomonas putida* KT2440, a saprophytic soil bacterium, which colonizes plant roots, is a suitable microorganism for the removal of pollutants and a stable host for foreign genes used in biotransformation processes (Bagdasarian and Timmis 1982; Moreno et al. 1988; Iwasaki et al. 1994; Jimenez et al. 2002). The *lcp* gene from *Streptomyces* sp. K30 was amplified employing the primers pqspBBR-for:_Sal and pqspBBR-rev:_Sac, and also the primers pqspJB-for:_Sbf and pqspJB-rev:_Sac (Table 1. Both PCR products were cloned into the *Sal*I/*Sac*I site of pBBR (Table 1) and the *Sbf*/*Sac*I site of pJB, yielding pBBR1MCS2::Lcp_His6 and pJB653::Lcp_His6, respectively. Although all experiments with conditions optimized for *Pseudomonas* strains resulted in the overproduction of Lcp in the supernatant and successful purification by nickel chromatography of the His-tagged protein, Lcp was inactive.

High-level expression and secretion of proteins in the native form has been proven to be difficult in both hosts, *E. coli* and in γ-Proteobacteria such as *P. putida*. *Escherichia coli* cells are the most commonly used host cells for large-scale production of recombinant proteins, but some proteins are difficult to express in *E. coli*. This includes proteins with low stability (Bertani 1951), proteins that are toxic to the host, and proteins that tend to form IBs. Due to the low content of *lcp* in *Streptomyces* sp. K30, it is difficult to isolate the overproduced protein from the original producer. Therefore, we applied a new strategy.

### Heterologous expression of *lcp* in *S. lividans* TK23, TK24, and *S. erythraea*

The transfer of *lcp* to and expression of Lcp in the different host strains described above had no effect on the activity of this protein. Alternative methods for the overexpression of *Streptomyces* proteins in engineered expression hosts of the same or related species of this genus were in the past successfully applied to the overproduction of different enzymes (Kayser and Kilbane 2001; Moreno et al. 2003, 2005; Hidalgo et al. 2004; Torres-Bacete et al. 2007; García-Hidalgo et al. 2011).

*Streptomyces lividans* TK23, TK24, and *S. erythraea* were chosen as expression hosts as the expression of Lcp activity in Gram-negative bacteria *E. coli* and *Pseudomonas* was not successful. In contrast to *S. lividans* TK23, the genome of *S. lividans* TK24 is completely sequenced and the genome has definitely no *lcp* homologous. Needless to say, we have analyzed TK23 for *lcp* homologous with PCR without evidence but the expression of Lcp in TK24 cannot be disputed, as there is no *lcp* homologous in the sequence of the genome.

**Figure 2:**
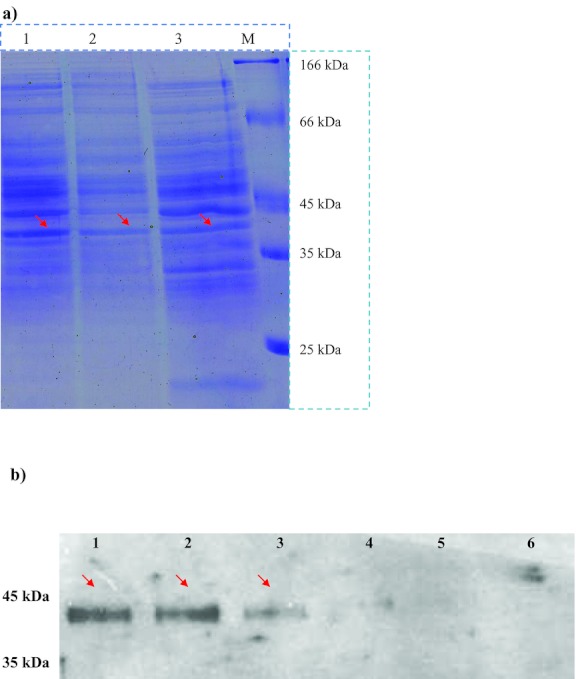
Immunological detection of Lcp from *S. erythraea* pIJ6021:: *lcp*, *S. lividans* TK23 pIJ6021:: *lcp*, and TK24 pIJ6021:: *lcp* by Western blotting. The expression of Lcp in *S. erythraea*, *S. lividans* TK23, and TK24 was analyzed by SDS-PAGE. All protein solutions were obtained from cell-free concentrated extracellular supernatants of cells of *S. erythraea*, *S. lividans* TK23, and TK24 grown in LB medium at 30°C for four days. **(a)** Electropherogram of an SDS-polyacrylamide gel after separation of the proteins. Proteins in the gel were stained with Coomassie brilliant blue R250. **(b)** Western blot employing anti-Lcp-IgGs prepared from an SDS-polyacrylamide gel. Lane 1: *S. erythraea* harboring pIJ6021:: *lcp*, lane 2: *S. lividans* TK24 harboring pIJ6021:: *lcp*, lane 3: *S. lividans* TK23 harboring pIJ6021:: *lcp*, the controls are lane 4: *S. erythraea* harboring pIJ6021, lane 5: *S. lividans* TK24 harboring pIJ6021, and lane 6: *S. lividans* TK23 harboring pIJ6021. The approximately 43-kDa Lcp protein recognized by the anti-Lcp-IgGs is marked with an arrow.

Therefore, *lcp* was cloned in the *E. coli*–*Streptomyces* shuttle expression vector pIJ6021. The resulting hybrid plasmid pIJ6021::*lcp* was transferred to other *Streptomyces* strains by protoplast transformation to study its expression. Bands presenting proteins of the expected size were visible in SDS-polyacrylamide gels after separation of the concentrated supernatants of the recombinant strains of *S. lividans* TK23 and TK24 as well as *S. erythraea* (Fig. 2a). Furthermore, Western blot analysis and immunological detection employing the Lcp antibodies raised against the purified Lcp protein of strain K30 confirmed the results and showed that Lcp was indeed synthesized by the recombinant strains (Fig. 2b).

**Figure 3:**
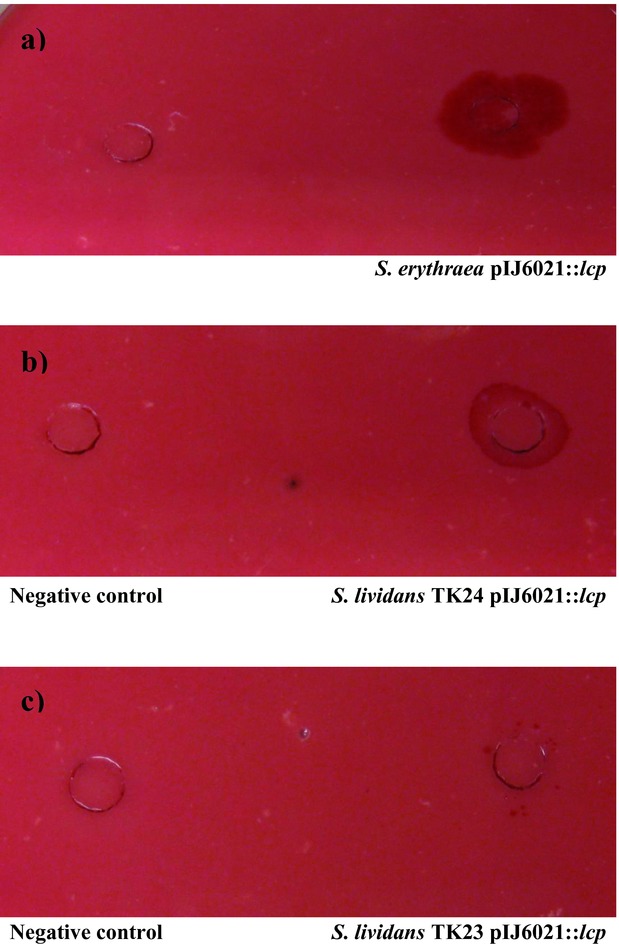
Effects of the experiment to demonstrate Lcp activity using the supernatant of Lcp-expressing strains on latex overlay agar plates. The concentrated supernatant (500 mL to 50 mL) of the mutants is shown on the panels. Both sides of the panels are furnished with the concentrated supernatant. Concentrated supernatant from (a) *S. erythraea* pIJ6021:: *lcp*, (b) *S. lividans* TK24 pIJ6021:: *lcp*, and (c) *S. lividans* TK23 pIJ6021:: *lcp* produces clear zones stainable with Schiff's reagent (right side). These strains obviously secreted a functional Lcp, as indicated by the formation of a halo. On the left, the negative control, harboring only pIJ6021 and producing no clear zones, is shown. After incubation for two to three days, agar plates were stained with Schiff's reagent to visualize aldehydes resulting from poly(*cis* -1,4-isoprene) cleavage.

As described previously (Jendrossek and Reinhardt 2003), purified NR latex from *H. brasiliensis* was used for the preparation of overlay agar plates to analyze the activity of Lcp. For this, MSM agar plates were covered with an overlay of MSM agar containing 0.2% (v/v) dispersed latex concentrate. These latex overlay agar plates were used to demonstrate clear zone formation and also growth of the recombinant strains. After four to seven days cultivation of the recombinant strains of *S. lividans* TK23, TK24, and *S. erythraea* harboring the plasmid pIJ6021::*lcp* on NR latex overlay plates at 30°C clear zones was observed. Thiostrepton (25 μg/mL) was used for plasmid maintenance. A recombinant strain harboring only the vector without *lcp* did not form clear zones. Furthermore, we have conducted an important experiment to demonstrate Lcp activity using the supernatant of these Lcp-expressing strains in vitro (Fig. 3a–c). All three strains obviously secreted a functional Lcp, as indicated by the formation of halo. This is the first time when Lcp activity using the supernatant of Lcp-expressing strains was successful. This is an important result for future works, for example, the difficult purification of Lcp.

### Deletion of Lcp from *Streptomyces* sp. strain K30

An additional experiment was necessary to verify the function of *lcp* in rubber degradation. The construction of a knock out mutant of *lcp* in *Streptomyces* sp. K30 was not successful hitherto; unfortunately, only very low transformation and conjugation frequencies were achieved with this newly isolated strain although intensive efforts were made to increase the transfer rates of foreign DNA. In this study, we succeeded in constructing a knock out mutant.

**Figure 4:**
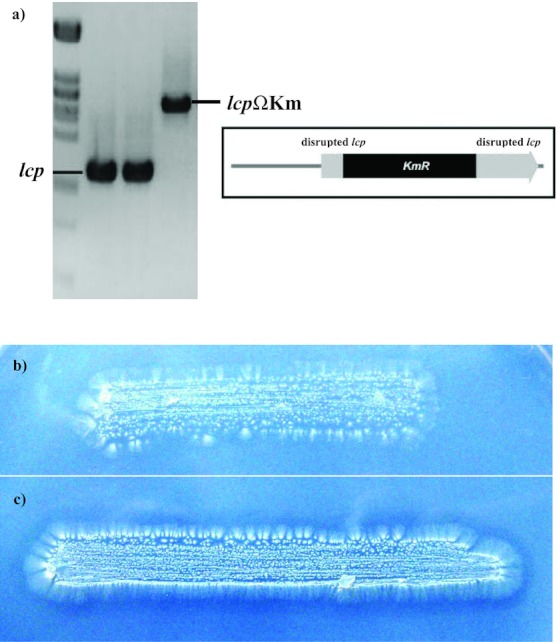
Analyses of *lcp* disruption mutants of *Streptomyces* sp. K30. **(a)** Screening for *lcp* disruption mutants of *Streptomyces* sp. strain K30 by colony PCR. Cell material from a single colony of a putative *lcp* disruption mutant was suspended in 50-μl TE buffer, and the suspension was then boiled for 15 min. After centrifugation, 0.5 μl was applied as template for a PCR employing the primers N_Lcp and C_Lcp (Table 1); the product was subsequently separated in a 1% (w/v) agarose gel. M, λ DNA digested with *Pst* I; WT, *Streptomyces* sp. K30 wild type; *lcp* ΩKm disruption mutant of *Streptomyces* sp. K30. **(b and c)** Effect of the knock out *lcp* mutant on clear zone formation by *Streptomyces* sp. K30. **(b)***Streptomyces* sp. K30 harboring *lcp* ΩKm and **(c)** the wild-type *Streptomyces* sp. K30 were cultivated for seven days on a natural rubber (NR) latex overlay agar plate at 30°C.

The 1224-bp sequence comprising the entire *lcp* coding region including a unique restriction site for *Sma*I was located downstream of the putative start codon. For this reason, this fragment was amplified by PCR using the primers N_Lcp and C_Lcp; subsequently it was cloned into pGEM-T Easy (Table 1), which does not possess a cleavage site for *Sma*I. The resulting plasmid, pGEM-T::*lcp*, isolated from *E. coli* TOP10 could not be digested with *Sma*I, indicating methylation at its recognition site, it was transferred to *E. coli* ET12567 lacking the DNA methylase. The plasmid DNA could then be linearized with *Sma*I, and an approximately 1000-bp *Sma*I-*Sma*I kanamycin resistance cassette (ΩKm) was inserted at position 281 of *lcp*. The 2.2-kbp *lcp*ΩKm DNA fragment was amplified by PCR, and the resulting linear DNA fragment was purified, dialyzed, and transferred to *Streptomyces* sp. K30 by protoplast transformation. Recombinant clones were selected for chromosomal integration of the *lcp*ΩKm fragment on St-I medium agar plates containing kanamycin (50 μg/mL). In total, many individual transformation reactions yielded more than 80 kanamycin-resistant colonies. Colony PCR using the primers N_Lcp and C_Lcp gave only one transformant that did not exhibit the wild-type 1224-bp PCR product, but the 2.2-kbp *lcp*ΩKm knock out PCR product instead (Fig. 4). All other clones exhibited both the wild-type *lcp* fragment and the 2.2-kbp *lcp*ΩKm fragment, indicating an unspecific integration of the 2.2-kbp *lcp*ΩKm DNA fragment somewhere else in the chromosome.

If Lcp has an essential function for poly(*cis*-1,4-isoprene) degradation in *Streptomyces* sp. K30, its absence should have a deleterious effect on the utilization of this polymer. The effect of *lcp* inactivation on growth of mutant *Streptomyces* sp. K30*_lcp*ΩKm in presence of poly(*cis*-1,4-isoprene) was abundantly clear. Even after two weeks of incubation, *Streptomyces* sp. K30_ *lcp*ΩKm no clear zone formation was observed; staining with Schiff's reagent the reaction was negative (Fig. 4b). Based on this result, the effect of Lcp on the utilization of the polymer was obvious. The capability of the *lcp* knock out mutant to use poly(*cis*-1,4-isoprene) as carbon source was compared to that of the wild type in mineralization experiments. Highest mineralization of poly(*cis*-1,4-isoprene) was obtained with the wild-type strain *Streptomyces* sp. strain K30. After 50 days of mineralization, the wild-type K30 had metabolized about 1.63% of the supplied NR cultures to CO_2_. In contrast, the *lcp* knock out mutant mineralized only about 0.82% of NR to CO_2_ in the same period. The experiment to measure the value of metabolized rubber was repeated three times with the wild-type *Streptomyces* sp. K30 and the *lcp* disruption mutant *Streptomyces* sp. K30_*lcp*ΩKm. The rubber degradation rate of the wild-type *Streptomyces* sp. K30 is quite slow (seven days to form clear zones on latex overlay agar plates), strains such as TK23 and TK24 show similar results; hence, we consider the difference to be significant.

### Complementation of *Streptomyces* sp. K30_*lcp*ΩKm

The genetic complementation of the *lcp* knock out mutant was analyzed in detail. Plasmid pIJ702::*lcp*_1, harboring the wild-type gene including the native promoter region of *lcp* from *Streptomyces* sp. strain K30, was transformed by protoplast transformation into the corresponding mutant. This plasmid restored the wild-type phenotype in the *lcp* knock out mutant. The recombinant strain was able to produce a clear zone on latex overlay plate and to produce aldehydes as revealed by staining with Schiff's reagent. These results confirmed the successful complementation of the *lcp* knock out mutant with the wild-type *lcp* gene.

This was the first time that an *lcp* knock out mutant from *Streptomyces* sp. strain K30 was successfully generated. All previous efforts in our laboratory had failed. In contrast to the parent strain (Fig. 4c), the *lcp* mutant was unable to form a clear zone (Fig. 4b). Furthermore, it did not form metabolites staining with Schiff's reagent. Moreover, mineralization experiments clearly revealed that poly(*cis*-1,4-isoprene) degradation was almost completely diminished in the *lcp* knock out mutant of *Streptomyces* sp. strain K30 when compared to the wild-type *Streptomyces* sp. strain K30. These indisputable findings confirmed that the initial cleavage of poly(*cis*-1,4-isoprene) is solely dependent on Lcp in *Streptomyces* sp. K30. It is therefore unlikely that other proteins than this are additionally involved in rubber cleavage of the poly(*cis*-1,4-isoprene) chain in this bacterium.

This study encourages further studies of rubber degradation in Gram-positive microorganisms. In the future, the latex-clearing protein, Lcp, must be purified to unravel the reaction mechanism of this enzyme acting on polyisoprene and to employ this protein for biotechnological applications, for example, for the conversion of rubber waste material.
